# Isolation of Enteric Nervous System Progenitor Cells from the Aganglionic Gut of Patients with Hirschsprung’s Disease

**DOI:** 10.1371/journal.pone.0125724

**Published:** 2015-05-18

**Authors:** David J. Wilkinson, George S. Bethell, Rajeev Shukla, Simon E. Kenny, David H. Edgar

**Affiliations:** 1 University of Liverpool Institute of Translational Medicine, Liverpool, United Kingdom; 2 Department of Pathology, Alder Hey Children’s Hospital NHS Foundation Trust, Liverpool, United Kingdom; 3 Department of Paediatric Surgery, Alder Hey Children’s Hospital NHS Foundation Trust, Liverpool, United Kingdom; Instituto Butantan, BRAZIL

## Abstract

Enteric nervous system progenitor cells isolated from postnatal human gut and cultured as neurospheres can then be transplanted into aganglionic gut to restore normal patterns of contractility. These progenitor cells may be of future use to treat patients with Hirschprung’s disease, a congenital condition characterized by hindgut dysmotility due to the lack of enteric nervous system ganglia. Here we demonstrate that progenitor cells can also be isolated from aganglionic gut removed during corrective surgery for Hirschsprung’s disease. Although the enteric nervous system marker calretinin is not expressed in the aganglionic gut region, *de novo* expression is initiated in cultured neurosphere cells isolated from aganglionic Hirschsprung bowel. Furthermore, expression of the neural markers NOS, VIP and GFAP also increased during culture of aganglionic gut neurospheres which we show can be transplantation into cultured embryonic mouse gut explants to restore a normal frequency of contractility. To determine the origin of the progenitor cells in aganglionic region, we used fluorescence-activated cell sorting to demonstrate that only p75-positive neural crest-derived cells present in the thickened nerve trunks characteristic of the aganglionic region of Hirschsprung gut gave rise to neurons in culture. The derivation of enteric nervous system progenitors in the aganglionic gut region of Hirschprung’s patients not only means that this tissue is a potential source of cells for future autologous transplantation, but it also raises the possibility of inducing the differentiation of these endogenous cells *in situ* to compensate for the aganglionosis.

## Introduction

During embryonic development, the enteric nervous system (ENS) is mainly derived from cells originating in the vagal region of the neural crest which migrate caudally to colonize the whole length of the gut [1]. During this migration through the mesenchyme of the gut wall, the cells behave as multipotent progenitor cells (ENSPC) as they proliferate and differentiate into the neurons and glial cells of ENS ganglia [2]. While some of the transcription factors, receptors, ligands and other cell signalling components necessary for ENS development have been established [3], the mechanisms determining ENSPC migration/stasis, proliferation/quiescence and differentiation of specific neural phenotypes are not fully understood.

ENSPC have been isolated from the gut of both embryonic and postnatal rodent embryos, and also from postnatal human bowel [4]. When cultured as neurospheres, ENSPC display ENS progenitor cell properties as they proliferate and differentiate to produce both glia and neurons with phenotypes characteristic of ENS neural cells [5,6,7,8,9,10]. Most importantly, when these neurospheres are transplanted into aganglionic gut their neurons have been demonstrated to be functionally active, restoring normal patterns of gut contractility [9,10]. However, it remains to be seen whether the ENSPC derived from postnatal gut are progenitors remaining after development, or alternatively if they arise from the de-differentiation of more mature postnatal ENS cells caused by the effects of isolation and culture [11,12].

The objectives of work on ENSPC are both to elucidate fundamental mechanisms of development and to facilitate translational research aimed at the development of clinical applications. Specifically, ENSPC present novel opportunities to treat Hirschsprung’s disease [4], which is characterized by absence of ENS ganglia in variable lengths of the distal gut in newborn children [13]. The aganglionic gut of Hirschsprung patients is usually removed by surgery, but there is significant rate of postoperative morbidity due to defective bowel function [14]. The reasons for poor outcomes are multiple. For example, extensive aganglionosis involves resection of significant amounts of distal gut that may result in significant morbidity due to short bowel syndrome. Additionally, one major factor is that current surgical techniques leave the aganglionic internal anal sphincter *in situ* [13]. Consequently, we have proposed that restoration of ENS neurons to the remaining sphincter would offer a postoperative adjunct therapy for Hirschsprung’s disease [14]. To this end, we have previously isolated and begun characterization of ENSPC from normally innervated regions of gut obtained from Hirschsprung patients, opening the way for autologous transplantation thereby avoiding the necessity of immunosuppression [10,15].

Before any such transplantation therapy is attempted, several crucial questions with regard to efficacy and safety need to be answered. For example, ENSPC proliferate readily in culture but we do not know if they will revert to a quiescent state after transplantation, or alternatively if will be necessary use differentiated cells derived from them for transplantation [16]. Furthermore, the origin and identity of cell(s) present in the heterogeneous populations of cells present in neurospheres must be established in order to optimize differentiation of the neuronal phenotypes necessary to regulate gut contractility [6].

To address these questions, we have begun to undertake an in depth study of the origins and behavior of ENSPC obtained from Hirschsprung patients. Surprisingly, we found that ENSPC-like cells can be isolated from the aganglionic region of Hirschsprung gut. Additionally, we demonstrate present evidence that ENSPC from the aganglionic gut arise from cells of the thickened extrinsic nerve trunks characteristic of the aganglionic gut regions in Hirschsprung’s disease. These observations have implications both for our understanding of ENS developmental mechanisms involved in the pathogenesis of Hirschsprung’s disease, and also for the development of novel therapies. Thus, cells from aganglionic Hirschsprung gut might be used for autologous transplantation, or alternatively neurogenesis might be stimulated within the aganglionic segment in situ, thereby removing the need for surgery.

## Materials and Methods

### Ethics statement

Ethical approval for the isolation of human ENS progenitor cells was given by the UK North West 3 Research Ethics Committee (Ref: 10/H1002/77). Written parental consent was obtained before samples were taken. Both ganglionic and aganglionic human gut specimens were obtained from 12 patients (age range 1–6 months) with short-segment Hirschsprung’s disease, and 1 patient with the long segment form of the disease. Frozen tissue sections were examined at the time of surgery by a clinical pediatric pathologist to establish the absence of enteric ganglia and the presence of thickened nerve trunks characteristic of affected bowel.

The use of mouse tissue in this study was approved by the Animal Welfare Committee of the University of Liverpool which is the Institutional Animal Care and Use Committee (IACUC). The University of Liverpool is a Licenced Establishment in accordance with the United Kingdom Animal (Scientific Procedures) Act of 1986. Pregnant mice were sacrificed by exposure to carbon dioxide gas in a rising concentration, followed by dislocation of the neck, followed by decapitation of 11.5 days post-coitum embryos, which are appropriate methods under Schedule 1 of the Act. The principle of refinement (Section 2A of the Act) requires that mice were humanely killed with a minimum of pain, suffering and distress, by a person with appropriate training, registered as competent to kill animals for this project according to Schedule 1 of the Act in the register kept by the Animal Welfare Committee (IACUC) of the University of Liverpool. The study did not require a Home Office project license because no in vivo experiments or regulated procedures as defined by the Act were carried out.

### Neurosphere culture

One cm^2^ full-thickness ganglionic and aganglionic human gut specimens were obtained from 12 patients (age range 1–6 months) with short-segment Hirschsprung’s disease, and 1 patient with the long segment form of the disease. Tissue for cell culture was transferred to the laboratory on ice, wrapped in sterile, saline-soaked gauze. Single cell suspensions were obtained from human ganglionic and aganglionic colonic biopsies as previously described [10,15]. Briefly, tissue was cut into 1mm^3^ pieces and placed in 1% (w/v) Dispase and 1% (w/v) Collagenase IV (both from Life Technologies, Paisley, UK) for a total of 2 h. The samples were triturated every 15 min using a 5ml pipette and the single cell suspensions were removed from remaining clumps after each trituration. The single cell suspensions were combined and centrifuged before resuspension in culture media. Typical yields from each specimen were 3–6 x 10^6^ cells.

To ensure optimal neurosphere culture conditions, we compared the culture medium previously used by our group and others (Dulbecco’s modified Eagle medium low (1% w/v) glucose, supplemented with 1% (v/v) fetal calf serum, 20 ng/mL FGF2 and 20 ng/mL EGF) with a medium lacking added growth factors consisting of Dulbecco’s modified Eagle medium (high glucose, 4.5% w/v), supplemented with 199 media (20% v/v), heat-inactivated horse serum (7% v/v), 100units/ml penicillin and 100 μg/ml streptomycin (all media components from Life Technologies). Between 5x10^5^ and 10^6^ cells dissociated from Hirschsprung ganglionic gut were plated into 6cm Nunc tissue culture dishes and incubated at 37°C in a humidified 5% CO_2_ incubator. Media were refreshed every 3 days by careful aspiration of 50% of the volume in each dish and replacement with the same volume of fresh media. Under these conditions neurospheres typically formed between 7–14 days. The loosely attached neurospheres were harvested every 14–15 days by detachment from the more strongly attached cells on the culture substratum by pipetting a stream of medium over them. The cells were then passaged be digesting the neurospheres with 0.05% (w/v) trypsin and mechanical dissociation by trituration to produce single cell suspensions for further couture to form secondary and tertiary neurospheres [15]. Assessment of cell phenotypes was performed on cells dissociated from neurospheres that had been allowed to attach to 8-well chamber slides (Thermo Scientific, Leicestershire, UK) previously coated with poly-D-lysine and laminin [15,16]. The neurospheres that developed during culture in the medium with horse serum had the overall appearance of those previously described after culture in the presence of growth factors which contained cells expressing p75 (S1 Fig). To make a quantitative comparison between the neurosphere cells grown in the two media, dissociated cells were counted with a hemocytometer. The counts show that there was an initial decrease in cell numbers, due to removal of the neurospheres from those cells present in the initial dissociates brought into culture that adhered strongly to the culture dishes (S1 Fig). Subsequently neurosphere cell numbers increased to the same extent in both media (S1 Fig). Furthermore the proportions of neurosphere cells expressing the neural crest cell marker p75 increased to similar extents in both media during culture (S2 Fig). Consequently, the subsequent experiments reported in this paper were carried out using cells cultured in the medium with horse serum but lacking growth factors. Furthermore, all comparisons of cells derived from ganglionic and aganglionic gut regions utilized biopsies derived from the same patient.

### Microscopy

Clinical diagnosis of all ganglionic and aganglionic bowel segments was established at surgery and routine H&E staining of frozen biopsy sections. The diagnosis was also confirmed after surgery by immunohistochemistry for calretinin on paraffin wax-embedded, 4 μm-thick sections of rectal biopsies (S3 Fig), using a monoclonal mouse antibody (Calret 1, Dako, Glostrup, Denmark) at a dilution of 1:400. After 30 min incubation, unmasking was carried out in pH 9 buffer at 120°C. Pure Envision dual link^®^ (Dako) was then applied for 30 min. Negative controls omitted the primary antibody and positive controls were histologically normal rectal biopsies. Paraffin-embedded sections from approximately half the cohort of patients were examined for calretinin staining, and in all cases this staining confirmed the original pathological diagnosis of Hirschsprungs aganglionic bowel made at surgery. Furthermore, there were no discrepancies between the clinical diagnosis of aganglionosis and the confirmatory observations described in this paper.

For immunofluorescence, samples for frozen tissue sections were fixed at time of surgery in 4% (w/v) paraformaldehyde and embedded in Shandon Cryomatix (Thermo Fischer Scientific). Seven μm serial frozen sections were prepared from full thickness colonic biopsies. Cells dissociated from fresh tissue or neurospheres were either spun down onto slides using a Shandon Cytospin3 (Thermo Scientific, Leicestershire, UK) or fixed in situ with 4% (w/v) paraformaldehyde after culture. Primary antibodies used for immunofluorescence were used at the following concentrations: rabbit anti-calretinin (Abcam ab702) 1:25; mouse anti- GFAP (Sigma-Aldrich G3893) 1:250; mouse anti-p75 (Abcam ab3125) 1:1000; rabbit anti-neuronal nitric oxide synthase (NOS, Abcam ab63602) 1:200; mouse anti-smooth muscle actin (SMA, Abcam ab7817) 1:200; rabbit anti-S100 (Abcam ab868) 1:500; mouse anti-β tubulin III (Tuj1, Covance MMS-435P) 1:1000; mouse anti-human specific ribonucleoprotein (HRNP, Merck Millipore 05–1508) 1:50. Alexafluor^®^ secondary antibodies (Life Technologies) were all used at 1:1000.

### Fluorescence activated cell sorting (FACS)

Single cell suspensions from fresh bowel samples were transferred to ice-cold FACS buffer (sterile PBS supplemented with 1% w/v bovine serum albumin, penicillin and streptomycin). 5 x 10^6^ cells were then incubated on ice for 30 min either with 1:1000 mouse anti-p75 antibody (Abcam ab3125) or isotype control antibodies in 200μl FACS buffer. Cells were then centrifuged and re-suspended in 200μl FACS buffer with 1:1000 Alexafluor 488^®^ goat anti-mouse secondary antibodies. After 45 minutes the cells were re-suspended in 1ml FACS buffer and sorted using FACSaria^®^ (BD Biosciences). Cell aliquots from p75-positive and-negative subpopulations were then cultured in poly-D-lysine/laminin-coated 8 well chamber slides (Thermo Scientific) for 6 days, before assessing neuronal differentiation by Tuj1 immunofluorescence.

### Neurosphere transplants and measurement of bowel contractility

Timed pregnant CD-1 mice were purchased from Charles River (UK) Ltd., (Margate, UK). The *ex vivo* assessment of gut contractility together with observation of cell migration and phenotype was as previously described using aganglionic distal colon explants dissected from 11.5 days *post coitum* CD-1 mouse embryos [10]. Positive ganglionic controls used explants of the same age but which extended to include the cecum that at this stage of development contains neural crest cells which migrate during explant culture [10]. Explants were cultured for 8 days alone as negative controls, or transplanted at the proximal end with a single 300–400μm diameter primary neurosphere derived from aganglionic Hirschsprung bowel. The frequency of bowel contraction was recorded and analyzed using Diamtrak software [10]. After measurement of contractility, the explants were prepared for immunostaining.

### Statistical analysis

GraphPad Prism^®^ 4.03 was used to analyse data. Non-parametric continuous data were analysed using Mann-U Whitney, a two-tailed t test was used for parametric data 1-way ANOVA analysis with Tukeys correction was used for parametric grouped data and a Krustal-Wallis test for non parametric data. Standard error of the mean was used for comparing outcomes of repeated experiments. P < 0.05 was taken as significant.

## Results

### Cell phenotypes in ganglionic and aganglionic Hirschsprung gut

**Fig 1:**
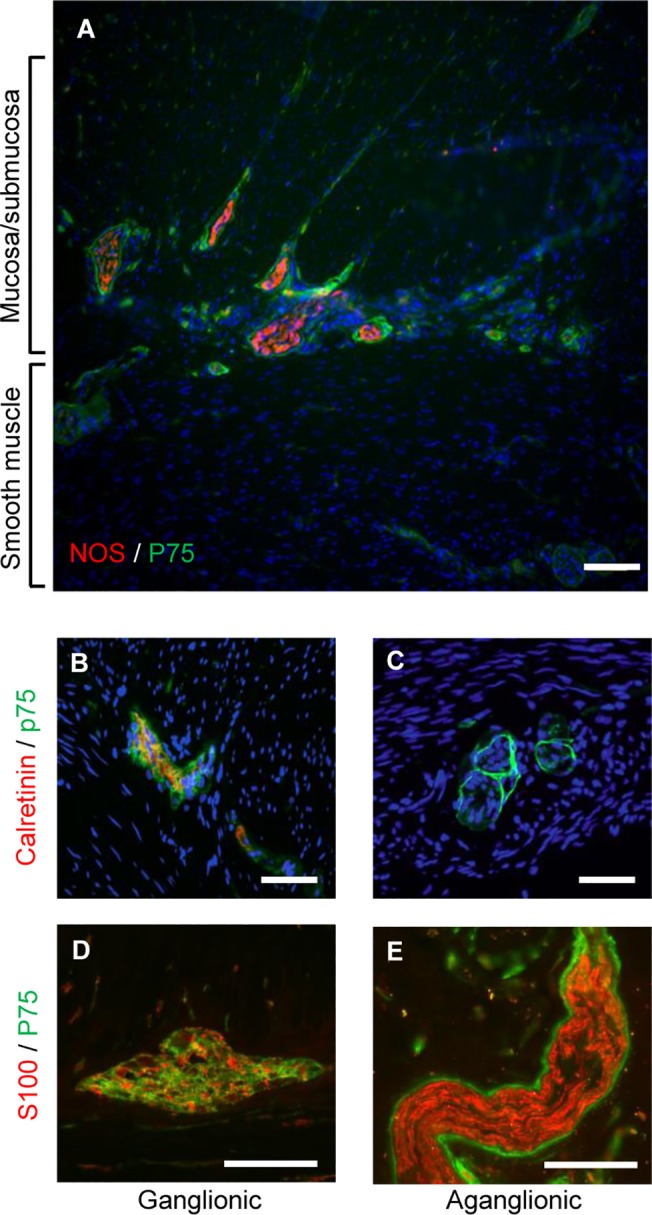
Expression of neural markers in colonic biopsies from Hirschsprung patients. A) Full thickness frozen section of aganglionic Hirschsprung bowel showing thickened nerve trunks immunolabeled for the neural crest cell marker p75 (green) and neuronal NOS (red). Note abundance of nerve trunks and fibers in the submucosal region, together with fewer thicker nerve trunks in running through the smooth muscle (C, E). B) Immunolabeling of frozen sections for calretinin expression (red) confirmed presence of ENS in ganglionic gut, and its absence in distal aganglionic gut (C). Antibodies to p75 also stained myenteric ganglia (green) in the frozen sections (B, D). The thickened nerve trunks of aganglionic gut displayed p75 immunoreactivity with very strong immunofluorescence in the perineurium and lower levels of immunoreactivity in the endoneurium (C, E). The glial cell marker S100 was also expressed (red) in ganglia (E) and endoneurium of the thickened nerve trunks of aganglionic gut (E). In contrast to p75 staining, there was no S100 immunoreactivity in the perineurium of the thickened nerve trunks (E). All sections are counterstained with DAPI and are typical of the 12 short segment Hirschprung’s tissues examined. Scale bars: A = 120μm; B—E = 100 μm.

The ganglionic and aganglionic regions of Hirschprung colon were confirmed by immunohistology of biopsies using the ENS marker calretinin which is absent from the aganglionic gut (see Fig 1 and S3 Fig, also [17,18]). The hypertrophic thickened nerve trunks, characteristic of aganglionic Hirschsprung bowel [19,20], were negative for calretinin (Fig 1C). Consistent with previous observations [20], the thickened nerve trunks were surrounded by cells expressing high levels of the p75 marker for neural crest-derived cells (Fig 1C and 1E). Cells expressing lower levels of p75 were also seen throughout the nerve trunks in the aganglionic region and within ganglia of the ENS in the ganglionic region (Fig 1B–1E), but the ganglia did not have surrounding cells with high levels of p75 (Fig 1B and 1D). Cells in both ENS ganglia and thickened nerve trunks also expressed the glial marker S100 (Fig 1D and 1E), but the peripheral cells of the thickened nerve trunks did not display S100 immunoreactivity, in contrast to their intense p75 staining (Fig 1E).

### Culture and differentiation of cells from Hirschsprung gut

**Fig 2:**
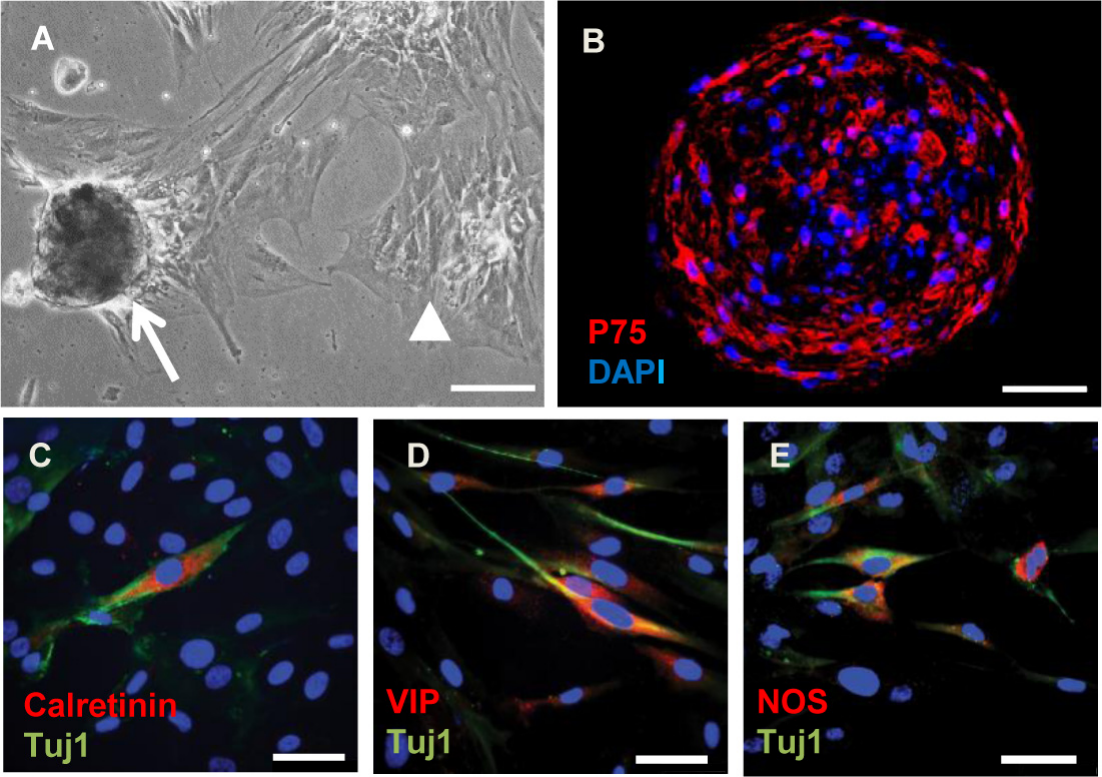
Appearance of neurosphere like-bodies and expression of neuronal markers by neurosphere cells derived from aganglionic Hirschsprung gut. A) Phase contrast photomicrograph of neurosphere-like bodies after 10 days culture. Arrow points to typical loosely attached neurosphere, and arrow head shows clusters of flat cells strongly attached to the substratum. B) Frozen section of neurosphere like body demonstrating cells immunofluorescent for p75 (red), nuclei counterstained with DAPI. C–E) Cells dissociated from the neurosphere-like bodies after 30 days culture followed by immunostaining (red) for calretinin (C), VIP (D), and neuronal NOS (E). The cells were also dual-stained (green) for the pan-neuronal marker Tuj1 and the nuclei counterstained (blue) with DAPI (B—E). Scale bars: A = 100μm; B = 50 μm and C—E = 20μm).

Cells isolated from all 12 short segment aganglionic Hirschsprung samples used in this study behaved similarly when brought into culture: during the first days of culture cells began to clump together to form loosely attached neurosphere-like bodies (Fig 2A), similar in appearance to the ENS neurospheres previously derived from normal ganglionic gut by ourselves and others [6,9,10]. Furthermore, frozen sections of these neurosphere-like bodies were used for immunofluorescence to demonstrate the presence of p75expressing cells (Fig 2B). To further characterize and quantitate these cells, neurosphere were dissociated and the single-celled suspensions allowed to attach to adhesive slides for immunofluorescence. Surprisingly, given the absence of ENS in the aganglionic gut, after 30 days culture some of the neurosphere cells derived from it had begun to express the pan-neuronal marker Tuj1 (Fig 2C–2E). Furthermore, subpopulations of these cells were positive for the ENS marker calretinin (Fig 2C), together with differentiated neuronal markers VIP (Fig 2D) and neuronal NOS (Fig 2E). Thus cells derived from aganglionic Hirschsprung gut lacking ENS neurons begin to express phenotypic markers characteristic of ENS neurons in neurosphere culture.

**Fig 3:**
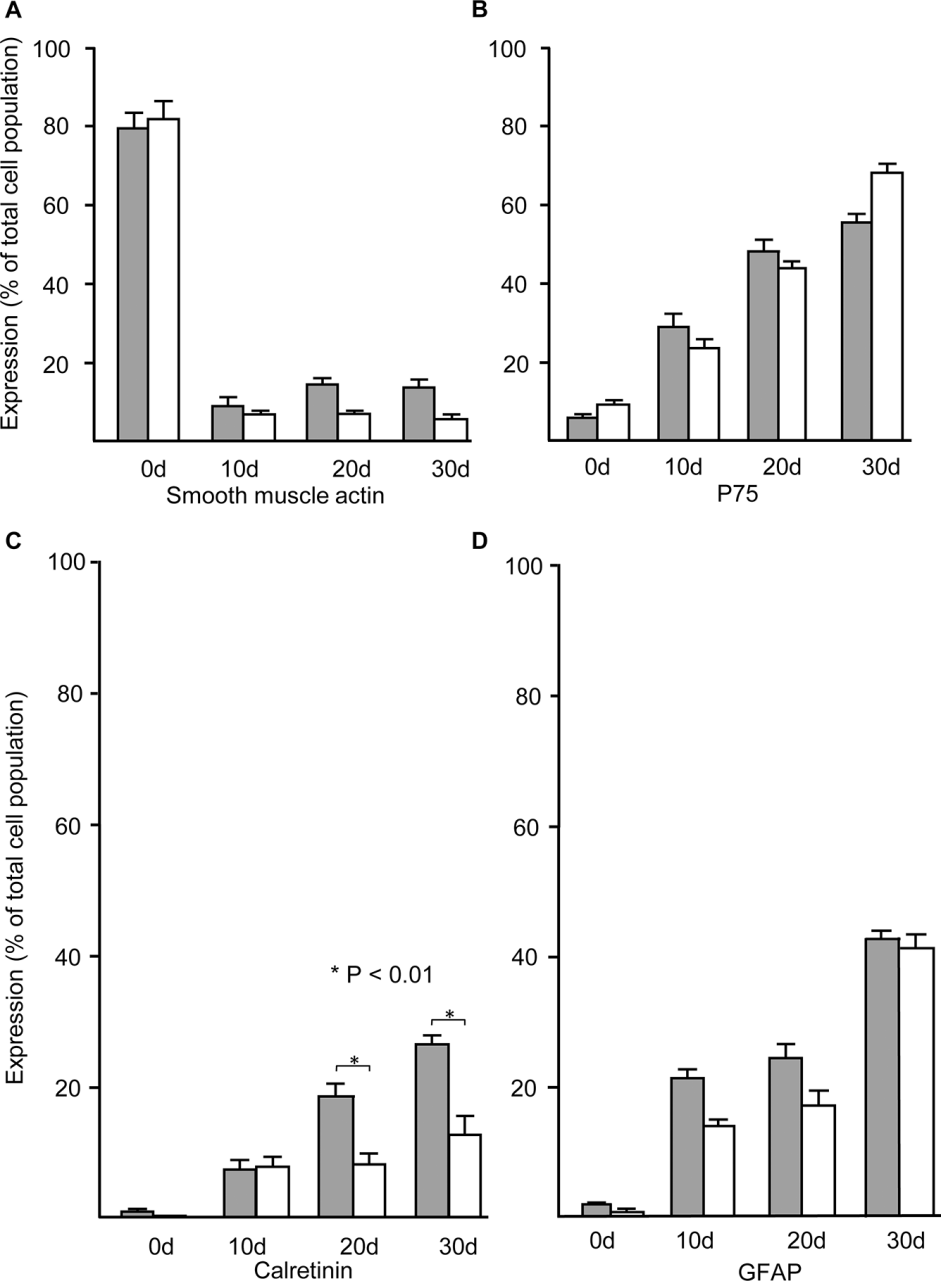
Time course of expression of cell-specific markers by cells immediately after dissociation from ganglionic and aganglionic Hirschsprung gut, and during neurosphere culture. Shaded columns show the percentage of cells in the populations derived from ganglionic Hirschsprung gut, and open columns the percentage from aganglionic Hirschsprung gut. The proportion of cells expressing smooth muscle actin (SMA) drops significantly immediately after isolation (0d) and remains low in the populations of cells dissociated from both ganglionic and aganglionic neurospheres (A). In contrast, expression of the neural crest marker p75 is low in initial isolates (0d) but increases over time until the majority of both ganglionic and aganglionic cells are p75 positive by 30 days (B). The percentage of cells expressing the ENS marker calretinin in isolates from ganglionic gut was very low (<2%) and undetectable in aganglionic isolates (C). However, after 1 day culture the proportion of calretinin positive cells increased in both ganglionic and aganglionic neurospheres, although after 20 days it remained significantly lower in the aganglionic neurospheres compared with ganglionic neurospheres (P<0.01). Low numbers of cells expressing GFAP were present in initial isolates of ganglionic and aganglionic gut, but their numbers increased gradually over the 30 day period of neurosphere culture (D). Error bars show SEM, n = 5 for both ganglionic and aganglionic cultures.

In order to characterize neural cell development in the cultures from aganglionic Hirschsprung gut tissue, we compared quantitatively the development of specific phenotypes from ganglionic and aganglionic gut cells immediately after initial dissociation and during culture as neurospheres. As expected, the majority of cells isolated directly from both ganglionic and aganglionic gut initially expressed smooth muscle actin, however, within the primary neurospheres harvested after the first 10 days of neurosphere culture their proportion dropped markedly during the first 10 days of neurosphere culture, and subsequently remained at low levels (Fig 3A). In contrast, the proportion of cells expressing the neural crest marker p75 was very low in freshly dissociated cells but rose during neurosphere culture so that the majority of cells in both ganglionic and aganglionic cultures were p75-positive by 30 days (Fig 3B). Reflecting the high levels of p75 staining by cells of the thickened nerve trunks (see Fig 1), there was initially a slightly higher proportion of cells expressing p75 in isolates from aganglionic relative to ganglionic gut, despite absence of the ENS (Fig 3B).

The specific ENS marker calretinin was initially only expressed by very low numbers of cells isolated from ganglionic gut, and as expected (see Fig 1) it was undetectable in the cells from aganglionic gut (Fig 3C). The number of calretinin positive cells from both ganglionic and aganglionic neurospheres increased markedly, although the proportion of aganglionic calretinin positive cells did not rise above 30% in ganglionic neurospheres, or 15% in aganglionic tissue (Fig 3C). The glial marker GFAP was expressed by a very low number of cells initially isolated from both ganglionic and aganglionic colon, but increased during neurosphere culture so that by 30 days after initial dissociation some 40% of both ganglionic and aganglionic gut derived cells expressed GFAP (Fig 3D).

### Fluorescence activated cell sorting and culture of p75 positive cells

**Fig 4:**
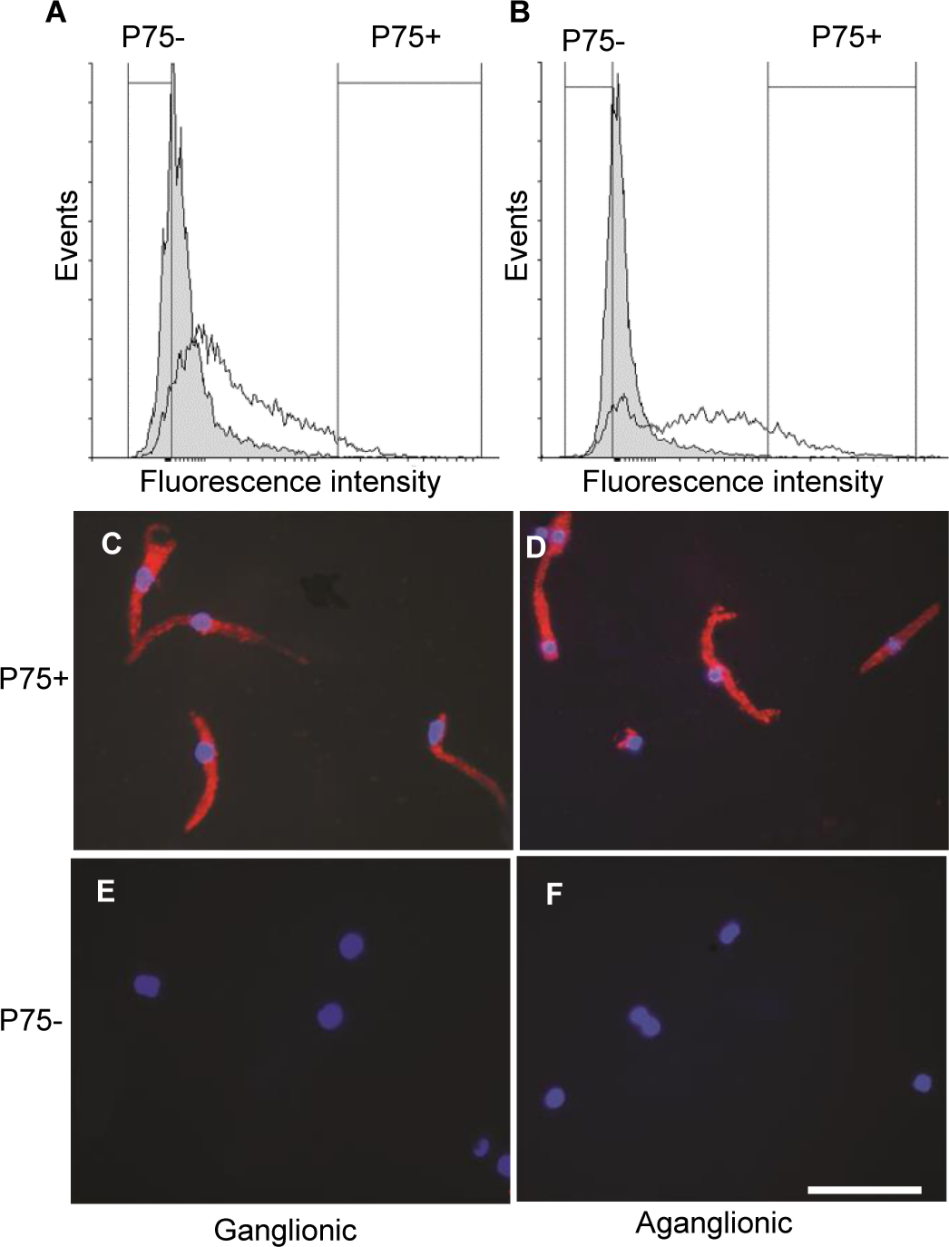
Expression of the pan-neuronal marker Tuj1 in cultures of freshly dissociated cells selected by FACS of p75 positive and negative cell subpopulations. FACS profiles showing numbers of p75 positive cells together with negative controls using isotype primary antibodies (shaded peaks). The range of p75- positive and-negative cells selected for culture from ganglionic gut (A) and aganglionic gut (B) are indicated by vertical lines. Tuj1 immunofluorescence staining of the p75 positive cell subpopulations from ganglionic (C) and aganglionic (D) bowel after 6 days culture. Note that the majority of cells are Tuj1-positive in both samples. Tuj1 immunofluorescence staining of the p75 negative cell subpopulations from ganglionic (E) and aganglionic gut (F). Note the absence of Tuj1 positive cells in both samples. Nuclei are counterstained with DAPI. Scale bar = 20μm.

Given the presence of p75 positive cells in the thickened nerve trunks of aganglionic Hirschsprung gut, we hypothesized that these are the precursors of the cells expressing neural markers found in increasing proportions in the neurosphere cultures (Fig 3). To demonstrate that this is the case, we used FACS to isolate subpopulations of p75-positive and-negative cells in the initial cell dissociates before culture. The FACS analysis confirmed that a minority of cells in the dissociates of both ganglionic (Fig 4A), and aganglionic (Fig 4B) Hirschsprung gut were p75 positive. Although the numbers of p75-positive cells from both samples were distributed with a continuous decrease of immunoreactivity down to background levels, the median for the distribution of p75 positive cell numbers from the aganglionic gut (Fig 4B) was noticeably higher than that for the ganglionic p75-positive cells (Fig 4A). To avoid mixed populations of p75 positive and negative cells in subsequent culture, the cell subpopulations selected for culture were taken from the highest and lowest regions of the p75 expression level profiles, which accounted for < 2% of cells in the isolates (see Fig 4A and 4B). After 6 days in culture, the majority of cells from the sorted P75 positive subpopulation of both ganglionic and aganglionic gut expressed Tuj1 (Fig 4C and 4D), whereas this neuronal marker was not detectable in cells derived from the p75-negative subpopulations (Fig 4E and 4F). Taken together, these results indicate that p75 positive cells present in the normoganglionic and aganglionic Hirschsprung gut can differentiate into neural cells in culture.

### Migratory behavior and functional potential of Hirschsprung aganglionic ENSPC transplanted into gut explants

**Fig 5:**
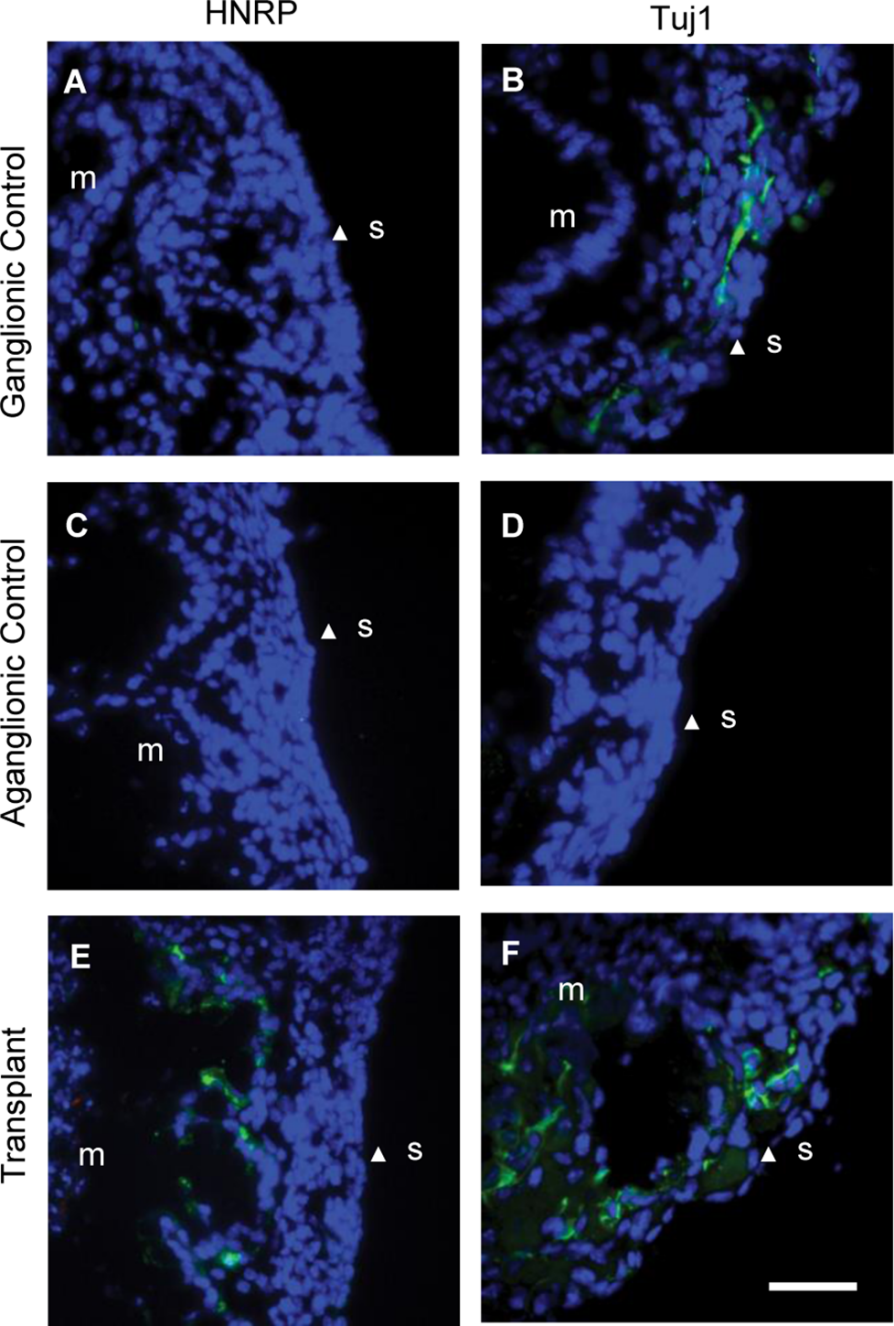
Migration of cells from transplanted neurospheres derived from aganglionic Hirschsprung gut in cultured explants of embryonic mouse colon. Immunofluorescent labeling with human-specific ribonucleoprotein (HRNP) antibody (green) (A, C, E). Human cells were absent in both control ganglionic and aganglionic explants (A, C). HNRP-positive cells had migrated into the developing smooth muscle of aganglionic mouse gut explant after transplantation with human neurospheres (E). Immunofluorescent labeling Tuj1 antibody (green) (B, D, F). Tuj1-positive cells cells were present in the ganglionic mouse gut explant reflecting its endogenous ENS (B). No Tuj1 immunoreactive cells were detected in the aganglionic mouse gut explant (D). Tuj1–positive cells were present in the mouse gut explant after transplantation of the human neurosphere (F). All images are orientated with the serosal surface (S, arrowheads) on the right, and the mucosal/submucosal region (M) of the explant on the left. Scale bar = 50μm.

To test the functional potential of neural progenitors derived from aganglionic Hirschprung gut, neurospheres were transplanted into the *ex vivo* aganglionic embryonic mouse gut explant model we have previously used to test cells from ganglionic gut [10]. The aganglionic status of the explants was demonstrated by the absence of Tuj1 staining (Fig 5D). However, after Hirschsprung aganglionic gut neurosphere transplant, cells expressing the human-specific cell marker HRNP had migrated away from the transplanted neurosphere into developing smooth muscle of the gut explants (Fig 5E). Furthermore, the initially aganglionic explant now contained cells expressing and the pan-neuronal marker Tuj1 (Fig 5F).

**Fig 6:**
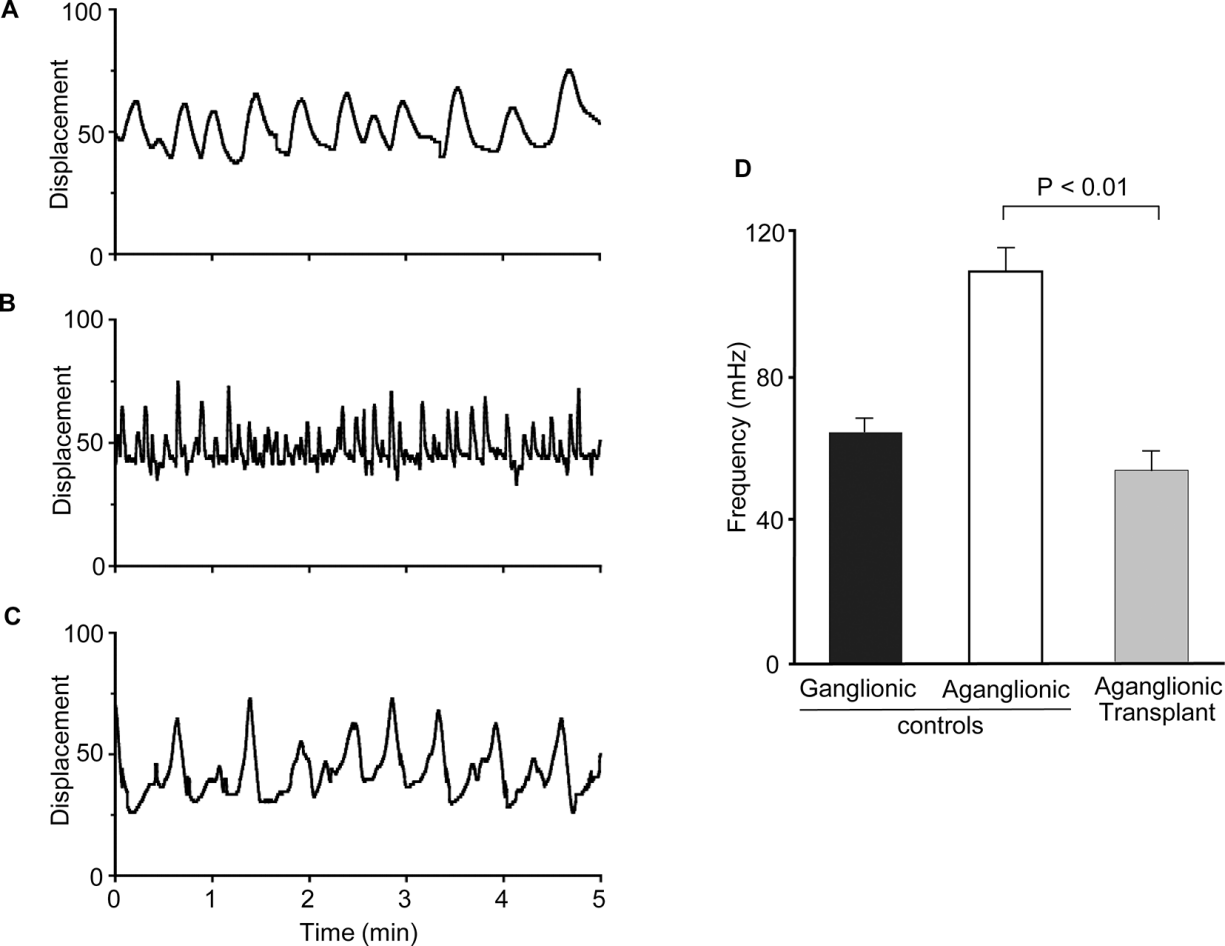
Effect of transplanted neurospheres derived from aganglionic human Hirschsprung bowel on the contractility of cultured embryonic mouse gut explants. Contractility was analysed after 8 days culture with/without neurosphere transplants using Diamtrak image analysis software. Ganglionic control explants displayed a slow, regular and uniform pattern of contraction (A). Aganglionic control explants displayed faster, and irregular contractility in comparison to the ganglionic pattern (B). Aganglionic explants after transplantation with a single neurosphere derived from human Hirschsprung aganglionic gut (C). Note that the pattern of contractility is now slower and more regular, similar to that of the ganglionic mouse explant (A). Single traces are shown which are representative of >5 separate experiments for each condition. Analysis of the frequency of contractions (D). Acquisition of data was gated to exclude small displacements less than 5% of the diameter of the gut wall. The contraction frequency of transplanted explants (C) was significantly lower (p<0.01) than that of untransplanted controls (B), but there was no significant difference between the slower contractions of ganglionic controls (A) and those of aganglionic gut explants after transplantation (ANOVA). Means ± SD are shown (n = 5).

The pattern of gut contractility was determined using a previously described optical motion capture technique [10]. Contractions in the aganglionic explants were rapid and irregular (Fig 6B) when compared with those in gut containing the endogenous ENS ganglia (Fig 6A). Significantly, the gut explants with aganglionic Hirschsprung neurosphere transplants displayed contractions of similar frequency to those of the endogenously innervated control, being about half that of the aganglionic gut control (Fig 6C and 6D). Taken together, these results demonstrate that neurospheres from the aganglionic gut of Hirschsprung patients can restore the frequency of gut contractility in this model system.

## Discussion

In this study we show that ENSPC-like cells can be isolated from the aganglionic gut of patients with Hirschsprung disease. The cells of neurospheres derived from the aganglionic gut are demonstrated to have the same phenotypes and functional properties as those previously described for ENSPC isolated from the ganglionic gut. Thus, they can differentiate into neuronal and glial cells [5,6,10], migrate through the gut wall [7,9,10], and restore normal patterns of contractility [9,10].

Aganglionic Hirschsprung bowel is characterized histopathologically not only by the absence of calretinin-positive ENS ganglia [18], but also by the presence of abnormal thickened extrinsic nerve trunks containing p75 positive cells [19,20]. The fact that only the p75 positive cells present in the aganglionic gut isolates give rise to cells expressing the pan-neuronal marker Tuj1 in culture demonstrates that the ENSPC originate from the thickened nerve trunks. These nerve trunks contain axons arising from neural crest-derived pelvic ganglia and S100-positive Schwann cells typical of immature peripheral nerves [21,22]. However, in addition to the p75-positive endoneurial Schwann cells, the peripheral cells of the trunks also express very high levels of p75 but which are negative for S100 (see Fig 1). Epineurial and perineural cells of peripheral nerves and ENS ganglia are derived from mesenchymal connective tissue [23], indicating that while the presence of cells expressing high levels of p75 at the periphery of the thickened nerve trunks is atypical of the ENS (see Fig 1), their location may be related to the migration of sacral neural crest cells along extrinsic pelvic axons which occurs both during normal development [22], and in aganglionic gut. [21].

The migratory properties of sacral neural crest cells differ from those of vagal cells [22,24], and it has recently been demonstrated that vagal (but not sacral) neural crest cell derivatives in the gut secrete extracellular matrix molecules that facilitate crest cell migration, and so may mediate interactions between vagal and sacral crest cells as they migrate in the gut [22,25]. It is therefore tempting to speculate that lack of vagal crest—derived cells in Hirschsprung distal gut results in the absence of the microenvironment necessary for the normal migration and/or development of the sacral progenitors as they migrate in along extrinsic fibres from pelvic ganglia, which in turn leads to the abnormal thickened nerve trunks in aganglionic Hirschsprung bowel.

It is now well established that the postnatal gut contains multipotent cells that are capable of differentiation into neurons and glia characteristic of the ENS [7,10,11,12,26]. The most obvious source of these cells would be expected to be the ENS itself, and this is supported by reporter gene-based lineage tracing studies demonstrating that cells expressing a glial phenotype can give rise to multipotent ENSPC in culture [11,12]. However, experiments *in vivo* using pulse-chase DNA labeling have provided evidence for the existence of a small population of extraganglionic cells able to colonize ganglia [27]. Our observations broaden these possibilities further by showing that in the absence of enteric ganglia in Hirschsprung gut, the p75 positive cells present in the thickened nerve trunks and isolated by FACS prior to culture give rise to ENSPCs within neurospheres.

Schwann and other glial cells have been well-documented to show a high degree of plasticity during development. Thus, it has recently been shown that neurons of parasympathetic ganglia develop from Schwann cell progenitors migrating from preganglionic nerves during development [28,29]. Furthermore, neurospheres containing multipotent cells can generated from Schwann cells of the sciatic nerve [30], and it has been shown that cells derived from cells expressing a phenotype characteristic of ENS glia proliferate and dedifferentiate to a multipotent state in culture, after which they can re-differentiate into neurons or glia [11,12]. Taken together, these observations are consistent with the hypothesis that the cells giving rise to ENSPC in neurospheres derived from Hirschsprung aganglionic gut are the p75-positive cells of the thickened nerve trunks. In support of this hypothesis, it should be noted that the very few patients with total intestinal aganglionosis lack such thickened nerve trunks [31], and we have been unable to isolate p75-positive cells or produce neurospheres from the gut of two such patients available to us (unpublished observations).

The fact that neuronal progenitors can be isolated from aganglionic Hirschsprung gut raises the possibility of using aganglionic tissue, normally discarded at surgery, to provide a source of cells for future autologous transplants in the treatment of Hirschsprung’s disease. Additionally, the existence of these cells raises the intriguing possibility to design future therapeutic modalities to stimulate neurogenesis in the aganglionic region *in vivo*, removing the need for surgery.

## Supporting Information

S1 Fig Neurosphere development in modified culture medium. A) Phase contrast photomicrograph of neurosphere-like cell aggregate derived from Hirschsprung gut after culture for 15 days in medium containing horse serum (see Materials and Methods for details). B) Immunofluorescence photomicrograph of frozen section through neurosphere-like aggregate after culture for 15 days in medium containing horse serum showing p75-positive cells. Scale bars: A = 50μm, B = 25μm. C) Cell numbers after neurosphere culture in growth factor-containing medium (GFM) or medium containing horse serum (HSM). Suspensions of cells were taken from freshly dissociated tissue and from aliquots of neurospheres removed from cultures at the times shown before trypsin digestion and trituration. Cell suspensions were counted with a hemocytometer, and cell numbers are expressed as a percentage of starting cell number in the initial tissue dissociates at time 0. Error bars show SEM, (n = 4 for all values). After the initial drop in cell numbers present in neurospheres after 15 days culture (due to removal of neurospheres from tightly adherent cells in the culture before dissociation and counting), cell numbers in cultured neurospheres increase markedly with time. However, there is no significant difference (P>0.25, two-way ANOVA) between cell numbers from GFM and HSM cultures at any of the individual time points.
(TIF)

S2 Fig Differentiation of p75- positive cells in neurospheres cultured in modified culture media. The percentage of cells expressing p75 are shown from neurospheres cultured with either growth factor medium (GFM) or horse serum medium (HSM), and in the initial cell dissociate at time 0. Aliquots of cultured neurospheres were harvested at the times shown and single cell suspensions prepared by trypsinization and trituration. The cells were then allowed to attach to tissue culture slides before paraformaldehyde fixation and processing for p75 immunofluorescence. Immunofluorescent cells were counted using a 40x objective by systematically surveying rows across the surface of the slide, corresponding to 25% of the culture surface area. Numbers of p75-positive cells are expressed as a percentage of the total number of cells counted, which had been counterstained with DAPI. There is a continuous increase in the number of p75 positive cells with time in culture but there is no difference (P>0.45) in numbers of positive cells between the two media at any single time point (ANOVA). Error bars show SEM, n = 4.
(TIF)

S3 Fig Expression of calretinin in colonic biopsies from Hirschsprung patients. The presence and absence of ENS ganglia in full thickness paraffin embedded sections of colonic biopsies of (A) ganglionic, and (B) aganglionic bowel was confirmed by immunohistology for calretinin after surgery. Sections are counterstained with hematoxylin/eosin. Scale bars = 100μm.
(TIF)
