# Supplementary figures and images for: Isolation of Enteric Nervous System Progenitor Cells from the Aganglionic Gut of Patients with Hirschsprung’s Disease
Source: PLoS One. 2015 May 18;10(5):e0125724. doi: 10.1371/journal.pone.0125724 (PMC4436257; doi:10.1371/journal.pone.0125724)

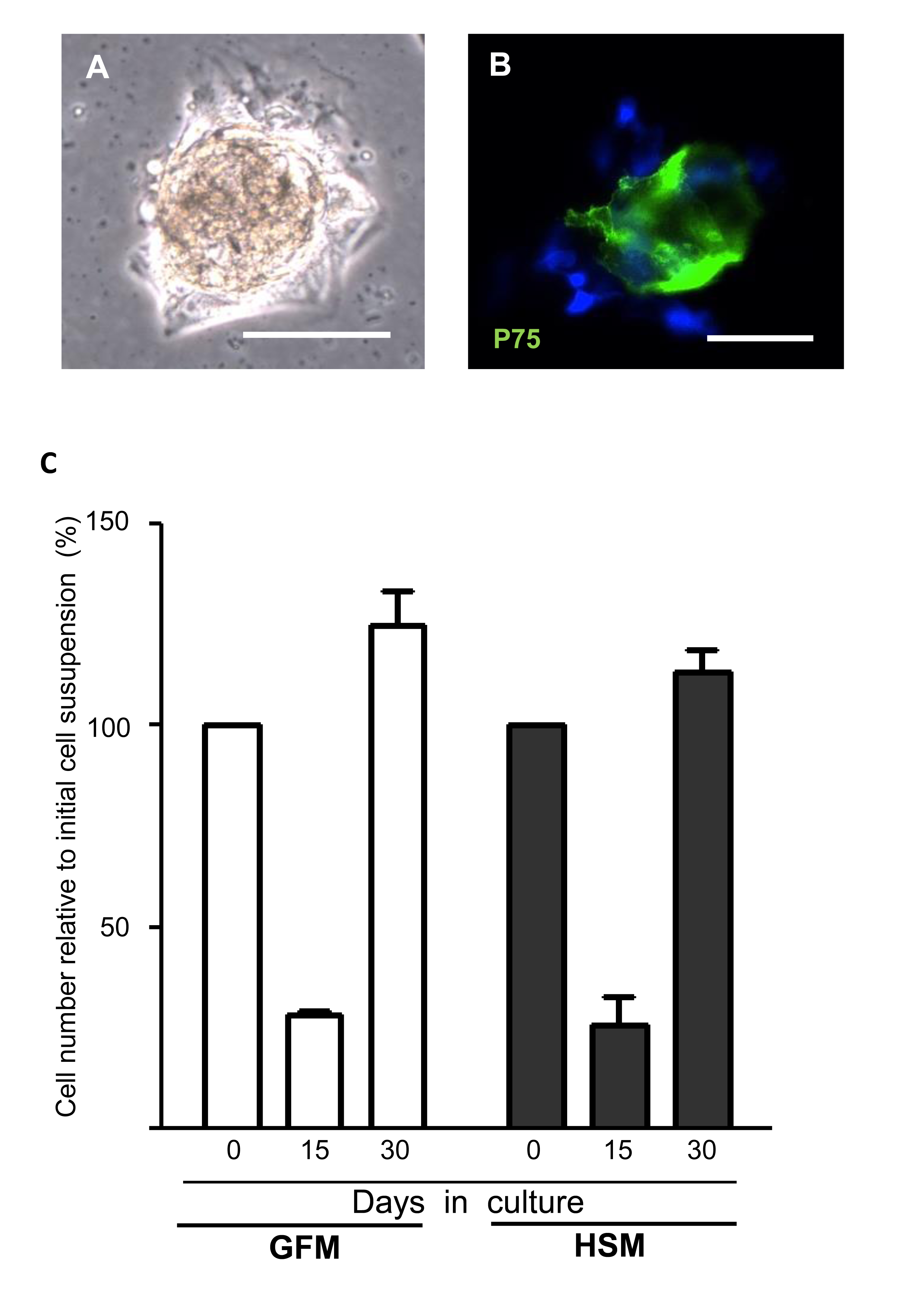

Supplement: S1 Fig — A) Phase contrast photomicrograph of neurosphere-like cell aggregate derived from Hirschsprung gut after culture for 15 days in medium containing horse serum (see Materials and Methods for details). B) Immunofluorescence photomicrograph of frozen section through neurosphere-like aggregate after culture for 15 days in medium containing horse serum showing p75-positive cells. Scale bars: A = 50μm, B = 25μm. C) Cell numbers after neurosphere culture in growth factor-containing medium (GFM) or medium containing horse serum (HSM). Suspensions of cells were taken from freshly dissociated tissue and from aliquots of neurospheres removed from cultures at the times shown before trypsin digestion and trituration. Cell suspensions were counted with a hemocytometer, and cell numbers are expressed as a percentage of starting cell number in the initial tissue dissociates at time 0. Error bars show SEM, (n = 4 for all values). After the initial drop in cell numbers present in neurospheres after 15 days culture (due to removal of neurospheres from tightly adherent cells in the culture before dissociation and counting), cell numbers in cultured neurospheres increase markedly with time. However, there is no significant difference (P>0.25, two-way ANOVA) between cell numbers from GFM and HSM cultures at any of the individual time points. (TIF) [file pone.0125724.s001.tif]

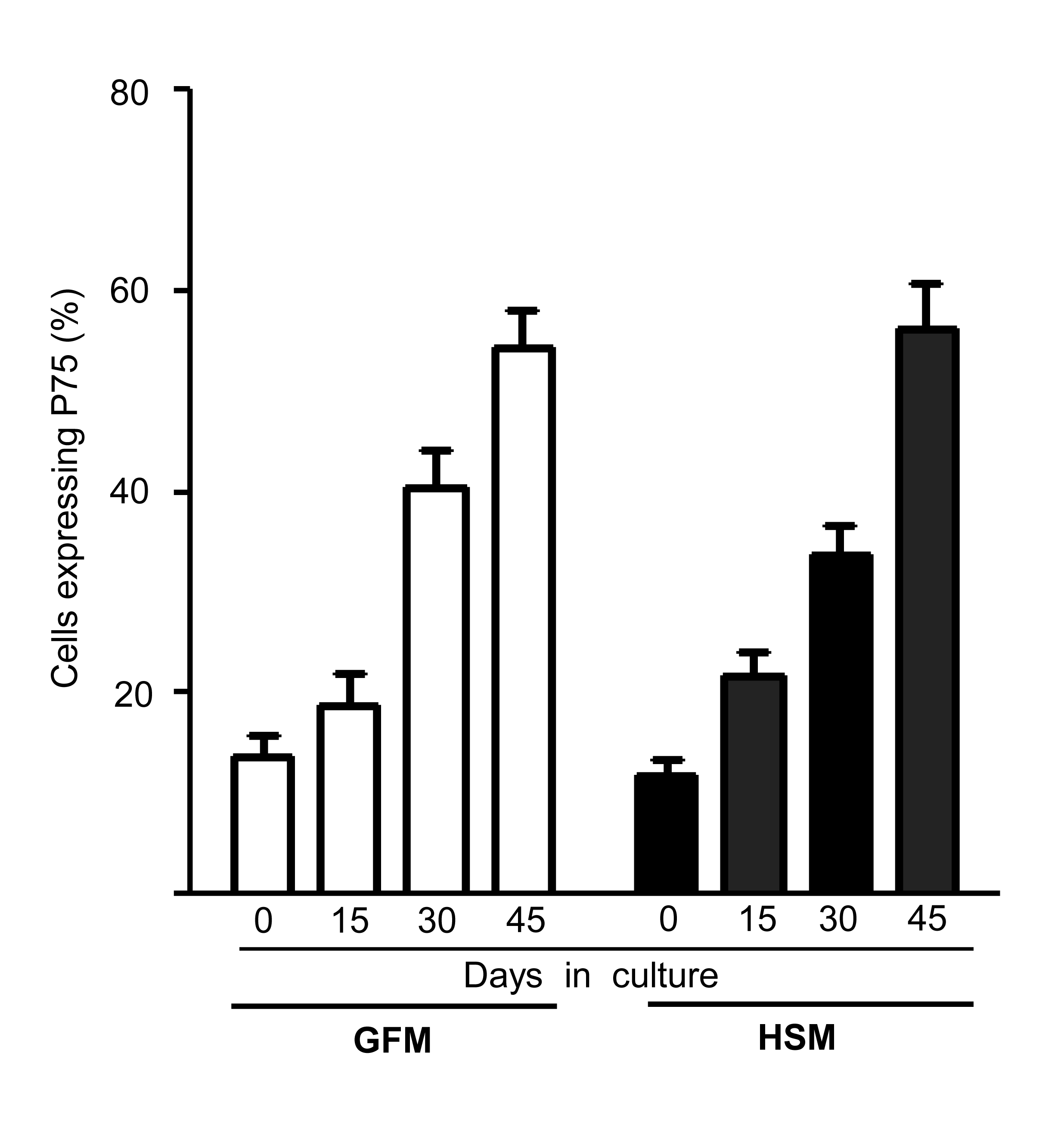

Supplement: S2 Fig — The percentage of cells expressing p75 are shown from neurospheres cultured with either growth factor medium (GFM) or horse serum medium (HSM), and in the initial cell dissociate at time 0. Aliquots of cultured neurospheres were harvested at the times shown and single cell suspensions prepared by trypsinization and trituration. The cells were then allowed to attach to tissue culture slides before paraformaldehyde fixation and processing for p75 immunofluorescence. Immunofluorescent cells were counted using a 40x objective by systematically surveying rows across the surface of the slide, corresponding to 25% of the culture surface area. Numbers of p75-positive cells are expressed as a percentage of the total number of cells counted, which had been counterstained with DAPI. There is a continuous increase in the number of p75 positive cells with time in culture but there is no difference (P>0.45) in numbers of positive cells between the two media at any single time point (ANOVA). Error bars show SEM, n = 4. (TIF) [file pone.0125724.s002.tif]

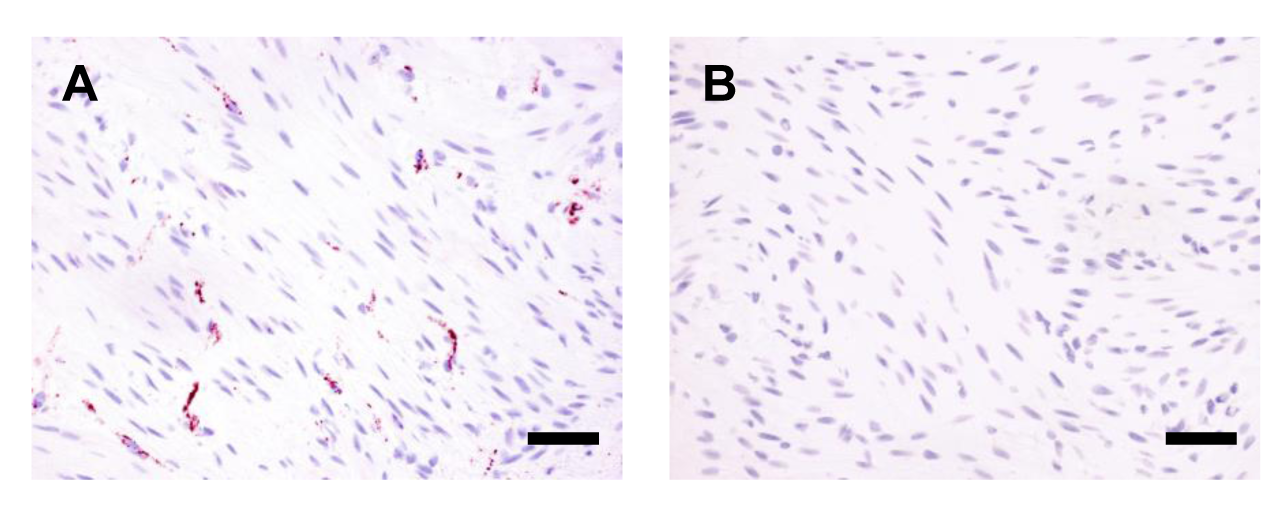

Supplement: S3 Fig — The presence and absence of ENS ganglia in full thickness paraffin embedded sections of colonic biopsies of (A) ganglionic, and (B) aganglionic bowel was confirmed by immunohistology for calretinin after surgery. Sections are counterstained with hematoxylin/eosin. Scale bars = 100μm. (TIF) [file pone.0125724.s003.tif]
